# A Rehabilitation Program for Individuals With Chronic Low Back Pain: Protocol for a Randomized Clinical Trial

**DOI:** 10.2196/31345

**Published:** 2022-10-31

**Authors:** Maíra Junkes-Cunha, Sofia Mendes Sieczkowska, Guilherme Torres Vilarino, Guilherme Bevilacqua, Alexandro Andrade

**Affiliations:** 1 Federal University of Pelotas (UFPel) School of Physical Education (ESEF) Pelotas Brazil; 2 Laboratory of Sport and Exercise Psychology Center of Health and Sport Science State University of Santa Catarina Florianopolis Brazil

**Keywords:** low back pain, chronic pain, therapeutic exercise, pain education

## Abstract

**Background:**

Low back pain (LBP) is the leading cause of disability worldwide. Physical exercise, as a treatment, is beneficial for the improvement of quality of life in patients with LBP, and it is widely accepted.

**Objective:**

We aimed to develop a protocol for a feasibility study that is designed to compare the effectiveness of different interventions in reducing pain, functional, and psychosocial factors among patients with chronic LBP after 8 weeks of randomization.

**Methods:**

This is a study protocol for a randomized controlled trial that will consist of individuals with chronic LBP who are aged between 18 and 65 years. Participants will be allocated, through block randomization, to one of the following groups: the motor control exercises (MCEs), pain education, MCEs+pain education, and usual care groups. The primary outcome will be pain intensity, and the secondary outcomes will be the pressure pain threshold, which will be measured with a digital algometer; LBP-related disability; fears and beliefs; the fear of movement; quality of life; mood states; and levels of depression and anxiety. The trial was approved by the ethics committee for research involving human beings of the Federal University of Pelotas (reference number: 5.717.390) in September 2022, and it will be conducted until August 2023.

**Results:**

The researchers are being trained to apply the questionnaires and carry out the interventions. Patient recruitment will begin at the end of 2022 and results are expected to be achieved by August 2023.

**Conclusions:**

Our trial will provide preliminary data regarding the feasibility and safety of MCEs and pain education for patients with LBP. It will also provide preliminary outcome data that can be used to identify the most efficient intervention and the level of health care that should be implemented in public health services.

**Trial Registration:**

Brazilian Registry of Clinical Trials U1111-1221-4106; https://ensaiosclinicos.gov.br/rg/RBR-2xx2r2/

**International Registered Report Identifier (IRRID):**

PRR1-10.2196/31345

## Introduction

### Background

Low back pain (LBP) is the leading cause of disability worldwide [1], resulting in significant economic burden for individuals, families, communities, industries, and governments [2]. LBP is deemed a chronic condition when the duration of continuous pain in the sacral, lumbar, or lumbosacral region exceeds 3 months. This may or may not be accompanied by irradiation to the lower limbs, which is called *lumbosciatalgia* [3].

Despite the high prevalence of LBP, many individuals prefer to modify their tasks at work rather than seek help due to the fear of losing their job [4]. However, these modifications may worsen the pain, as they can trigger other symptoms. Studies have shown that biopsychosocial factors, such as catastrophization, kinesiophobia, anxiety, depression, stress, job dissatisfaction, and financial concerns, may be associated with LBP [5-7].

Chronic pain treatment through physical exercise is beneficial for the improvement of patients' quality of life, and it is widely accepted. Studies have demonstrated positive results with strength training, walking, and stretching practices, such as pain reduction and improvements in depression and sleep quality [8]. In addition, there is evidence indicating that a multidisciplinary, biopsychosocial rehabilitation approach is more effective than an isolated approach for the treatment and management of chronic LBP [9].

Among the recommended exercise practices, it has been noted that motor control exercises (MCEs) are widely used for the reduction of recurrent LBP [10]. These exercises involve the voluntary contraction of deep muscles, especially the transverse abdominis and multifidus muscles, and aim to promote the activation of trunk stabilizing muscles to reduce symptoms and improve mobility [11,12]. The prescription and development of these exercises should be gradual and individualized, respecting the pain threshold of each patient in order to reduce the functional disability and catastrophization of the individual [13].

Despite research advances in LBP treatment, further studies that compare the effects of different treatment approaches are warranted to gain better understanding and develop intervention strategies that may be applicable to individuals with chronic LBP. There is currently no consensus about the effectiveness of treatments that account for the pain, functionality, and psychological factors in these individuals. Furthermore, few studies present their intervention protocols in detail, making it difficult to apply their obtained results in clinical practice. Therefore, we aim to provide detailed intervention protocols for MCEs and health that are reproducible and serve as a reference for interested professionals.

### Goal of the Study

The primary aim of our study is to analyze the effectiveness of a program that includes MCEs and pain education. The secondary aims are (1) to clarify the effects of the program on pain outcomes, including the subjective intensity of pain and the pressure pain threshold, and compare the MCEs group (M-group) and MCEs+pain education group (MP-group) to the pain education group (P-group) and usual care group (U-group), the P-group and MP-group to the M-group and U-group, and all 4 groups; (2) to clarify the effects of MCEs on physiologic and psychologic stress measurements and compare the U-group to the M-group, the U-group and P-group to the M-group and MP-group, and all 4 groups; and (3) to clarify the effects of the pain education intervention on physiologic and psychologic stress measurements and compare the U-group to the P-group and U-group and the M-group to the P-group and MP-group.

## Methods

### Study Design

**Figure 1:**
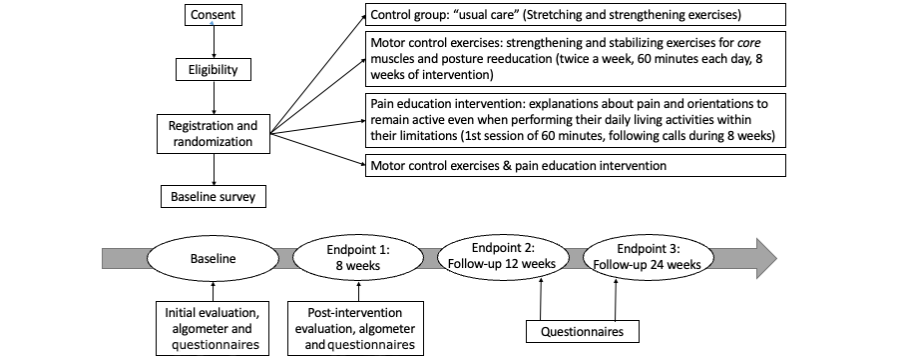
Flowchart of the study design.

Our study will be a parallel-group randomized controlled superiority trial with 4 arms, as shown in Figure 1. The target sample size is 900 individuals with LBP, who will be randomized (1:1:1:1 allocation) via computer-generated randomization. The groups will receive their assigned intervention according to the following allocations: (1) M-group, (2) P-group, and (3) MP-group; the U-group will receive the usual care intervention. Individuals will be instructed to not perform any form of physical exercise during the intervention period. We will compare the three types of interventions to determine their relative superiority to usual care.

### Ethics Approval

The study protocol was registered in the Brazilian Registry of Clinical Trials (registration code: RBR-2xx2r2; universal trial number: U1111-1221-4106). This protocol was created according to the SPIRIT (Standard Protocol Items: Recommendations for Randomized Trials) 2013 guidelines checklist [14].

Our research was approved by the ethics committee for research involving human beings of the Federal University of Pelotas (reference number: 5.717.390) in September 2022, and it will be conducted until August 2023.

### Recruitment

We will recruit individuals with LBP at outpatient clinics of basic health units in Pelotas, Brazil, while they wait for an evaluation. During recruitment, we will determine their study eligibility based on the inclusion and exclusion criteria in Textbox 1.

Individuals will be randomly allocated to intervention groups, having the same probability of being allocated to a group that will be exposed to the test interventions or the control group.

**Inclusion and exclusion criteria.**

**Inclusion criteria**

- History of low back pain in the last 6 months
- Pain intensity of ≥3 (Numeric Pain Rating Scale) during movement in at least one direction (flexion, extension, or rotation)
- Aged between 18 and 65 years

**Exclusion criteria**

- Previous history of rheumatic disease, lumbar myelopathy, tumors, or central or peripheral neurological disorders
- History of major trauma, fractures, or surgery in the lumbar region or signs of nerve root compression (major muscle weakness affecting the lower limbs, reduction or abolition of patellar and calcaneal reflexes, and decreased sensitivity in lower limb dermatomes)

### Intervention Program

The physiotherapists who will participate in the clinical trial will undergo prior training to standardize the interventions.

The interventions will be divided into the following groups:

- Group 1 (MCEs): individuals will perform a strengthening and stabilization exercise program for core muscles (stabilizers of the lumbar, pelvic, and abdominal muscles and the diaphragm muscle) and postural correction.
- Group 2 (pain education): individuals will receive a postural care guide and will be encouraged to remain active, performing their daily living activities within their limitations.
- Group 3 (MCEs+pain education): individuals will receive intervention through physical exercises and pain education.
- Group 4 (usual care intervention): individuals will undergo the usual treatment through the use of resources and the application of conventional techniques.

### The MCE Program

The purpose of the MCE program is to normalize the difficulty in the motion domain for each participant. The exercises will be divided into 3 stages of training. A progressive increase in the level of difficulty will occur as the patients correctly perform the exercises with ideal movement patterns (Table 1). A detailed anamnesis and physical examination will be performed to account for the condition of each individual participant. Additionally, these will allow us to determine the levels and goals for each stage of exercise and the progression time for each difficulty level.

In the first stage, individuals will start to learn how to perform isometric contractions of the transverse abdominis and multifidus muscles in the prone, supine, and quadrupedal positions. To ensure the correct activation of the transverse abdominis, it will be emphasized to participants that the lower part of the anterior abdominal wall (ie, below the level of the umbilical scar) should be pushed toward the spine to activate the muscle. Participants will be instructed to progressively increase the waiting time and number of contractions to up to 10 contraction repetitions×10 seconds of sustainment. At the first appointment, patients will receive awareness-raising advice for performing the deep muscle contractions and instructions on how to activate the aforementioned muscles, so that they can sustain the contractions during the exercises for about 30 to 45 minutes throughout the appointments. The patients will advance to the next stage when they show the ability to perform the exercise and control pelvic and lumbar movements with minimal effort while moving their upper and lower extremities.

In the second stage, the complexity of the exercises will be increased by progressing to functional exercises that involve performing trunk and upper limb coordination while maintaining trunk stability [15]. Patients will be taught to perform abdominal cocontraction in different postures and control movements of the lumbopelvic region with minimal effort while performing functional activities.

After learning all of the exercises in stages 1 and 2, participants will be instructed to perform trunk coordination exercises and weight loading for the upper limbs (ie, while maintaining the stability of the trunk) and consider the activities that presented difficulty or the activities for which they reported pain during implementation in the initial evaluation.

The last 10 minutes of appointments will be reserved for returning to baseline through free walking and stretching exercises.

**Table 1:** Progression of the exercises in the different stages of the motor control exercises.

	Phase 1	Phase 2	Phase 3
Progression 1	Prone position (10 repetitions×10 seconds of sustainment)	Supine position with alternating hip flexion Bridge exercise (isometric hip lift; 10 repetitions×20 seconds of sustainment)	Gym ball and dumbbell lifting in supine positiona
Progression 2	Supine position (10 repetitions×10 seconds of sustainment)	Quadrupedal position with alternating shoulder flexion and hip extension	Gym ball or dumbbell lifting in sitting and standing positionsa
Progression 3	Quadrupedal position (10 repetitions×10 seconds of sustainment)	Squats (20 repetitions) Elevation of upper limbs in sitting and standing positions (20 repetitions)	Hold weight in hands while simultaneously performing activities (sitting, standing, walking, and climbing stairs)^a^

^a^Progression will be determined according to the patient's functional condition.

### Pain Education

The educational program will be based on the protocol described by Traeger et al [16] and will consist of a biopsychosocial approach to dealing with fears and clarifying behavioral beliefs about pain and movement. Additionally, the program will explain the influence of emotional symptoms (stress, anxiety, kinesiophobia, etc) on chronic pain; the influence of lack of sleep and physical activity on LBP, gradual exposure to physical activity, and daily movements; and the neurophysiology of pain. The program will include the following three major components: (1) clarifying any useless beliefs about the nature of LBP, (2) presenting key concepts of the biology of pain, and (3) evaluating patients’ understanding and discussing recovery. Participants who are randomized to the pain education intervention will participate in two 1-hour sessions of pain education that will be conducted by a physiotherapist. The physiotherapist will be appropriately trained for the study and will be contacted weekly for clarifications on doubts and possible further guidance. Multimedia Appendix 1 shows the 3 steps of the Pain Education Program.

### MCEs and Pain Education

Individuals who are allocated to both interventions will receive guidance regarding the educational program for 1 hour during the first appointment, before participating in the MCE program for 8 weeks. The topics that will be covered throughout the treatment will be reinforced, emphasizing the factors for which individuals have more difficulty with changing their behavior.

### Usual Care Intervention

Conventional intervention will be based on exercises that are usually performed in clinical practice. It will involve the active stretching of the posterior thigh muscles (hamstrings) by using a resistance band. The therapeutic exercises will include (1) isometric exercises that activate the muscles of the core region and stabilizers of the loin-pelvic region and hips (the transverse abdominis and multifidus muscles, oblique and rectus abdominal muscles, gluteus minimus, and gluteus maximus); (2) isotonic exercises for the lower limbs (hip abduction and adduction and hip flexion and extension); (3) exercises that focus on movement functionality, which will be augmented by breathing and body perception techniques, body realignment, and muscular and joint imbalance correction; (4) resistance exercises that involve using an elastic band to strengthen lower limb musculature; and (5) guidance for performing daily life activities and explaining the importance of staying active to prevent recurrence or increases in pain, which will be given spontaneously as an initiative of the responsible physiotherapist.

The exercise interventions will be conducted with groups of 3 patients, and the program will last for 8 weeks. Patients will receive care twice per week, totaling 16 visits. The patients cannot perform any other types of exercises during the study period. To maintain patients’ compliance with the program, researchers will contact them each week to inquire about the progress of the interventions.

### Data Collection

The professionals (physicians, nurses, physiotherapists, psychologists, and physical education professionals) from the health network of Pelotas City, Rio Grande do Sul, Brazil, will refer patients to the program after assessing their eligibility. Patients who show an interest in participating in the program will be marked for an initial evaluation, during which the patients will be informed about the research procedures, evaluations, interventions, and duration. After obtaining all of the necessary information, patients who wish to participate must sign the informed consent form. Only researchers will have access to the database. To maintain data confidentiality, patients will be identified by numbers.

**Figure 2:**
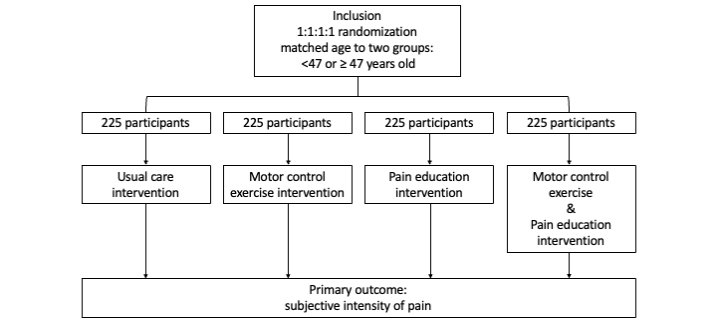
Study protocol.

The study protocol is shown in Figure 2. A baseline survey will be conducted after the allocation. Participants will receive questionnaires, including questions on demographic data and baseline data for outcomes.

### Outcome Measures and Assessment Points

Evaluations will be carried out before and after 8 weeks of intervention and the 12- and 24-week follow-ups. Individuals will be monitored weekly during the study period, and messages will be sent through WhatsApp (Meta Platforms) to collect information about pain intensity.

#### Primary Outcome Measure

The primary outcome measure will be a report of pain intensity on the Numerical Pain Rating Scale [17].

#### Secondary Outcome Measures

The secondary outcome measures will be as follows:

1. Comparison of disability outcomes
  - Scores measured by using the Roland Morris Disability Questionnaire [18]
2. Comparison of physiologic and psychologic stress
  - Score for the Fear Avoidance Belief Questionnaire [19]
  - Score for the Tampa Scale of Kinesiophobia [20]
  - Score for health-related quality of life from the Short Form-12 Health Survey Version 2 [21,22]
  - Score for the Brunel Humor Scale [23]
  - Scores measured by using the Beck Depression Inventory [24] and Beck Anxiety Inventory [25]
  - Scores measured by using the Pittsburg Sleep Quality Index [26] for evaluating sleep quality and behavior

Any adverse effects that are observed or reported by the patients will be recorded and considered for the final study outcomes. Furthermore, patients who experience adverse effects will be sent to a physician for adequate treatment.

### Study Procedures

#### Sample Size Calculation

Sampling will be probabilistic, and patients will be extracted from the LBP waiting list. The required sample size was calculated by assuming a mean difference of 1.5 and an SD of 2.4 [27]; the study requires a sample size of 225 per group, that is, 900 individuals in total.

#### Random Allocation and Blinding

After the initial evaluation, the participants will be allocated, through block randomization, to receive different interventions, which will be conducted by physiotherapists. Individuals who fulfill the inclusion criteria will receive clarification about the objectives and the procedures that will performed in the research, and their consent will be requested by asking them to sign the informed consent form, of which the terms were approved by the ethics committees of the Federal University of Pelotas and the Health Department of Pelotas City.

The participants will then be referred to the physiotherapist who will be selecting the interventions according to the groups to which the participants will be allocated. The individuals who are allocated to the M-group, MP-group, and U-group will participate in 16 treatment sessions (2 sessions per week, totaling 8 weeks of treatment) with an average duration of 40 minutes.

It will not be possible to blind the physiotherapists who are responsible for the interventions due to the type of research that will be conducted. However, evaluators and patients will be blinded to the treatment groups. Nonblinding conditions will only occur in cases of health emergencies.

### Statistical Analysis

The data will be analyzed by using SPSS version 20.0 (IBM Corporation). Descriptive statistics (means, SDs, and frequencies) will be used to present the characteristics of the participants, and inferential tests will be used to analyze the differences among the groups. Analyses will be performed based on intent-to-treat principles. The outcomes (Numeric Pain Rating Scale, Roland Morris Disability Questionnaire, Fear Avoidance Belief Questionnaire, Tampa Scale of Kinesiophobia, Short Form-36 Health Survey, Brunel Humor Scale, Beck Depression Inventory, Beck Anxiety Inventory, and Pittsburg Sleep Quality Index scores and the pressure threshold for pain) will be analyzed by using generalized estimation equations to compare the M-group, P-group, MP-group, and U-group at baseline, after 8 weeks, and at the 12- and 24-week follow-ups. The effects of groups, time, and group interactions with time (group×time) will be analyzed. The Bonferroni post hoc test will be used to identify the differences among the means of all variables. Missing data will be handled via multiple imputation [28]. The α established as the level of significance will be *P*<.05 (95%) for all hypothesis tests. The effect sizes will be checked by using the Hedges g test. The magnitudes of effect sizes will be interpreted as *trivial* (<0.2), *small* (0.2-0.6), *moderate* (0.6-1.2), *large* (1.2-2.0), *very large* (2.0-4.0), and *perfect* (>4.0) [29]. To represent the effect of the intervention, the delta (∆) of the analyzed variables (∆ = x ®postintervention − x ®preintervention) will be calculated.

## Results

The trial was presented to the municipal authorities and its practical applications were discussed in August 2022. The researchers are being trained to apply the questionnaires and carry out the interventions. Patient recruitment will begin at the end of 2022 and results are expected to be achieved by August 2023.

## Discussion

### Study Overview

Some studies have identified the positive effects of using a biopsychosocial approach in interventions for patients with chronic pain [9]. There is evidence that motor exercises reduce LBP through the activation of the deep lumbopelvic musculature [10].

In contrast to other randomized controlled trials, our study will investigate the effects of the association between motor exercises and pain education and compare them to the effects of an exclusively biopsychosocial approach.

The structure of our study reflects a scenario of actual clinical practice, and it will investigate the effects of the interventions on pain reduction after 8 weeks of intervention. A follow-up at 3 months after randomization will be performed to obtain information on pain and other clinical factors from the patients.

The results of our clinical trial study will give us information on the most efficient intervention strategies that will be implemented in a public health service within Brazil, which may be expanded to other physiotherapy centers and serve as a basis for planning health actions with the greatest impact on the population of people with LBP.

Future studies could explore the use of complementary modalities, such as mindful movements, among patients with chronic pain to identify the promotion of well-being.

### Study Implications

The structure of our study reflects a real scenario of actual clinical practice in a public health service. Its application may benefit many patients who have never been treated. The study proposal also offers a detailed exercise protocol and pain education approach that can serve as a reference for many professionals. Further, the identification of more appropriate intervention strategies for individuals with chronic LBP may result in changes in professional conduct and more treatment tools with a greater clinical impact.

### Conclusions

Our trial will provide preliminary data regarding the feasibility and safety of MCEs and pain education for patients with LBP. It will also provide preliminary outcome data that can be used to identify the most efficient intervention and the level of health care that should be implemented in public health services.

## Appendix

Multimedia Appendix 1 Pain Education Program.

## Abbreviations

LBP: low back pain
M-group: motor control exercises group
MCE: motor control exercise
MP-group: motor control exercises+pain education group
P-group: pain education group
SPIRIT: Standard Protocol Items: Recommendations for Randomized Trials
U-group: usual care group
