# Correction: A Rehabilitation Program for Individuals With Chronic Low Back Pain: Protocol for a Randomized Clinical Trial

**DOI:** 10.2196/44067

**Published:** 2022-11-17

**Authors:** Maíra Junkes-Cunha, Sofia Mendes Sieczkowska, Guilherme Torres Vilarino, Guilherme Bevilacqua, Alexandro Andrade

**Affiliations:** 1 Federal University of Pelotas (UFPel) School of Physical Education (ESEF) Pelotas Brazil; 2 Laboratory of Sport and Exercise Psychology Center of Health and Sport Science State University of Santa Catarina Florianopolis Brazil

In “A Rehabilitation Program for Individuals With Chronic Low Back Pain: Protocol for a Randomized Clinical Trial” (JMIR Res Protoc 2022;11(10):e31345) the authors noted 3 errors.

1. In the originally published article, the Methods section of the Abstract appeared as follows:

The trial was approved by the Human Research Ethics Committee in September 2018.

This has been corrected to:

The trial was approved by the ethics committee for research involving human beings of the Federal University of Pelotas (reference number: 5.717.390) in September 2022, and it will be conducted until August 2023.

2. In the originally published article, the Results section of the Abstract appeared as follows:

The trial was funded in 2018. Patient recruitment will begin at the end of 2021, as it involves patients who are on the waiting list of a public service and requires the health manager's permission to start the data collection, considering the current health scenario. Results are expected to be achieved by August 2022.

This has been corrected to:

The researchers are being trained to apply the questionnaires and carry out the interventions. Patient recruitment will begin at the end of 2022 and results are expected to be achieved by August 2023.

3. In the originally published article, the Results section appeared as follows:

The trial was funded in 2018 and presented to the municipal authorities. Its practical applications were discussed in August 2022, and the data collection process was structured to include the updating of patient contacts. Patient recruitment will begin at the end of 2022, as it involves patients who are on the waiting list of a public service and requires the health manager's permission to start the data collection, considering the current health scenario. Results are expected to be achieved by August 2023.

This has been corrected to:

The trial was presented to the municipal authorities and its practical applications were discussed in August 2022. The researchers are being trained to apply the questionnaires and carry out the interventions. Patient recruitment will begin at the end of 2022 and results are expected to be achieved by August 2023.

The correction will appear in the online version of the paper on the JMIR Publications website on November 17, 2022, together with the publication of this correction notice. Because this was made after submission to full-text repositories, the corrected article has also been resubmitted to those repositories.

