# Supplementary material for: A Rehabilitation Program for Individuals With Chronic Low Back Pain: Protocol for a Randomized Clinical Trial
Source: JMIR Res Protoc. 2022 Oct 31;11(10):e31345. doi: 10.2196/31345 (PMC9664319; doi:10.2196/31345)
Supplement: Multimedia Appendix 1 [file resprot_v11i10e31345_app1.docx]

## Appendix 1

PAIN EDUCATION PROGRAM

**Phase (i) Clarify any useless beliefs about the nature of lower back pain**

The physiotherapist will identify any useless beliefs, those that have been found to be associated with poor recovery from LBP, such as poor recovery expectations, intentions to avoid activity due to fear of damage and beliefs concerning a dependence on passive treatment approaches. These beliefs will be addressed by discussing any potentially unnecessary diagnostic, prognostic or therapeutic conclusions that the patient might have made. For example, a patient may express concern about a ‘disc slipping out’ with bending tasks at work. This belief will be identified to the patient as understandable, but inaccurate and unhelpful. Less threatening, evidence-based information will then be provided about the nature of the intervertebral dis, its inability to ‘slip’ and its relationship to LBP. The strength and stability of spinal structures will be emphasized.

**Phase (ii) Present key concepts of pain biology**

The key aspects of pain biology will be introduced and is designed to complement phase (i) as well as explanations given by the primary care provider. Pain will be presented as being a protective output of the brain that is influenced by many factors, rather than being a signal of tissue damage. More specifically, participants will be taught that: nociceptive input is modulated at the tissues, spinal cord and brain; the brain evaluates many inputs before selecting a response; pain is the conscious part of the response. This explanation provides support for current guideline instructions. For example, instructions such as ‘hurt is not the same as harm’, ‘stay active’ and ‘return to activities as soon as possible’, will be discussed in the context of evidence from pain biology.

In summary, the key concepts to be presented by the pain education are:

1. Pain is a protective mechanism, not necessarily a symptom of damage

2. In acute LBP, the system can become overprotective (sensitisation)

3. How one makes sense of their pain is an important factor for recovery

**Phase (iii). Evaluate understanding and discuss recovery**

The final component of the intervention reinforces the concepts outlined in phases (i) and (ii) and discusses recovery within these concepts. Understanding the cause of the symptoms and their variable relationship to tissue damage is discussed as the most important starting point for a good recovery. Emphasis is placed on the reliability of the tissue healing process, and the necessity of gradually returning to all activities. The explanation of pain biology in phase (ii) provides evidence that rehabilitation approaches such as moving the body are safe and effective. The participant is encouraged to discuss more specific aspects of rehabilitation (eg, set the first short-term goal) with their primary care provider.
